# GGT5 Is an Independent Prognostic Biomarker in Stomach Adenocarcinoma

**DOI:** 10.1155/2022/9983351

**Published:** 2022-02-26

**Authors:** Yong Huang, HaiLang Zhou, Junwei Zou, Dong Wang

**Affiliations:** ^1^Department of Gastrointestinal Surgery, The Second Affiliated Hospital of Wannan Medical College, Wuhu, Anhui, China; ^2^Department of Gastroenterology, Medical Center for Digestive Diseases, Lianshui People's Hospital Affiliated to Kangda College of Nanjing Medical University, Huaian, Jiangsu, China; ^3^Department of Hepatobiliary Surgery, The First Affiliated Hospital of Wannan Medical College, Wuhu, Anhui, China

## Abstract

Gastric cancer is one of the cancers with the highest incidence in the world. Gamma-glutamyltransferase 5 (GGT5) is expressed in different cancers and its role in cancers remains unclear. In this study, we aimed to evaluate the value of GGT5 in stomach adenocarcinoma (STAD). In TCGA, patients with high GGT5 expression had poor overall survival (*P*=0.006). Based on GSE62254, high expression of GGT5 was associated with poor OS (*P*=0.014) and PFS (*P*=0.042). The same result was observed in GSE14210. We further discovered that GGT5 expression was associated with stage, grade, and T stage. Further prognostic analysis of GGT5 showed that GGT5 was associated with prognosis in both univariate analysis (*P*=0.032) and multivariate analysis (*P*=0.029). We used gene set enrichment analysis (GSEA) to explore the possible mechanism of GGT5. GSEA suggests that overexpression of GGT5 may be involved in leukocyte transendothelial migration, JAK-STAT signaling pathway, MAPK signaling pathway, and melanoma. The high-expression GGT5 group had higher concentrations of M2 macrophages, T cell regulators, and monocytes, but the contents of plasma cells and M1 macrophages were higher in the low-expression GGT5 group. The results showed that the ESTIMATEScore, ImmuneScore, and StromalScore of the high-expression GGT5 group were higher than those of the low-expression GGT5 group. PD1 and CTLA4 expression levels were higher in the high-expression GGT5 group. The high-expression GGT5 group may be more effective for immunotherapy. These results suggested that GGT5 could be a potential prognostic molecular predictor in STAD.

## 1. Introduction

According to global cancer statistics, STAD is regarded as kind of the fatal tumors which is harmful to the safety and health of human beings, ranking fifth in the list of most normal cancers worldwide, and it is in the top three fatal elements of cancer-connected causes [1]. In the last decades, the death rate of this kind of illness has been obviously reduced. Given the advance and the research of society and medical technology, the progress of living standards would help reduce the prevalence of *Helicobacter pylori* (the main cause of gastric cancer) [2,3]. Despite advances in understanding the biology of gastric cancer, surgery or endoscopic resection is still the main treatment goal. But the 5-year mortality makes people worry about it [4]. Recently, immunotherapy and targeted therapies, such as immunoassay inhibitors against PD-L1, have shown a prospect to improve the prognosis of patients with unstable microsatellite cancer [5]. However, just a few people can get advantages from STAD [6]. On the whole, the identification of new biomarkers is essential for the effective treatment of STAD patients.

As an enzyme combined with membrane outside the cell, *γ*-glutamyltransferase (GGT) is concluded in the hydrolysis reaction of glutathione (GSH) *γ*-glutamate bonds and other *γ*-valeryl compounds. GGT5 is included in the group of GGT protein, which is a *γ*-glutamyl cell membrane protein capable of hydrolyzing GSH plus S-conjugates [7]. It matters a lot in medicine metabolism, redox management, and immune effect in vivo [8]. Many previous studies have suggested that GGT5 may promote inflammation [9–11]. One study showed that GGT5 was helpful to make tumors get bigger and resisted drug performance in lung cancer [12]. But the connection between GGT5 and clinical and prognostic pathological data of STAD is still unknown by people.

Through this research, we attempted to use the available parameters of TCGA to investigate the differential expression of GGT5 in patients with STAD and its relationship with clinical parameters.

## 2. Materials and Methods

### 2.1. Cell Lines Culture

The human gastric cell lines (BGC-823, AGS) and the normal control cell line (GES-1) were purchased from the Suzhou Medical University (Suzhou, Chinese). They were cultured in RPMI-1640 (Gibco, USA) at 37°C under a 5% CO_2_ atmosphere, complemented with 10% fetal calf serum (Gibco, USA), 1% streptomycin, and penicillin.

### 2.2. Quantitative Real-Time PCR, Western Blot (WB), and Immunohistochemistry (IHC)

We extracted total RNA with TRIzol reagent (Invitrogen) from cell lines (AGS, BGC-823, GES-1) according to the manufacturer's instructions. Then real-time quantitative PCR (qPCR) was conducted to detect the expression of GGT5. We used glyceraldehyde-3-phosphate dehydrogenase (GAPDH) as an endogenous reference. The primers used were as follows:

  GAPDH forward, 5′-GAAGGTGAAGGTCGGAGTC-3′;
  GAPDH reverse, 5′-GAAGATGGTGATGGGATTTC-3′;
  GGT5 forward, 5′-CAACACACCCTTTGGAGCGA-3′;
  GGT5 reverse, 5′-AAGTTGGGCTCGTACTCCAC-3′.

qPCR was performed on Roche LightCycler 96 using UltraSYBR mixture (Cwbio, China) under standard PCR conditions. We used western blot analyses to determine the protein level of GGT5 from gastric tissues according to the manufacturer's instructions. The antibodies of GAPDH and GGT5 were purchased from Thermo Fisher (USA). We also performed immunohistochemistry (IHC) experiments under the manufacturer's instructions. Our study was approved by the Ethics Committee of the Second Affiliated Hospital of Wannan Medical College, and informed consent was also acquired from patients.

### 2.3. Data Collection

We collected the gene expression data and the corresponding clinical characteristics data of gastric cancer from The Cancer Genome Atlas (https://portal.gdc.cancer.gov/), and 407 TCGA samples were also collected, which had 375 cases of stomach cancer and 32 common cases. 317 cases of stomach-related samples were full of clinical data. We used the Limma package to look for differentially expressed genes (DEGs); the adjust. *P* < 0.05 and |fold change (FC)|  > 2 was set as DEGs cutoff criterion. We also downloaded chip sequencing data sets (GSE13861, GSE13911, and GSE19826) from Comprehensive Gene Expression Omnibus (GEO) database (https://www.ncbi.nlm.nih.gov/geo/). There were 65 primary gastric adenocarcinoma cases and 19 surrounding normal tissues in GSE13861 [13]. The array data of GSE13911 consisted of 38 GC tissues and 31 adjacent tissues [14]. GSE19826 contained 12 paired GC tissues, 12 adjacent tissues, and 3 normal tissues [15]. We used the adjust. *P* < 0.05 and |FC|  >  1.5 as cutoff criterion to get DEGs.

### 2.4. Genes Expression Profiles

Next, the database TIMER2.0 online website was used (http://timer.cistrome.org/) to analyze the expression of GGT5 in 33 tumors [16]. The statistical significance calculated by the Wilcoxon test was shown by the number of ^*∗*^(^∗∗∗^: *P* value <0.001; ^∗∗^: *P* value <0.01; ^*∗*^: *P* value <0.05). We explored the expression of GGT5 in gastric cancer tissues and normal tissues in the TCGA database, GSE13861, GSE13911, and GSE19826.

### 2.5. Survival Analysis

An online website, KMP (Kaplan-Meier Plotter), is used to analyze, discover, and verify survival biomarkers. Data resources contain the completed GEO, which focuses on gene expression (https://www.ncbi.nlm.nih.gov/geo/), TCGA, and EGA (European Genome-phenomenon Archive) (https://ega-archive.org/) [17]. We used Kaplan-Meier Plotter to perform survival analysis based on gene expression level and hypothesis assessment using the log-rank test. We used Kaplan-Meier Plotter to explore the relationship between GGT5 expression and prognosis in gastric cancer (including GSE62254 and GSE14210). In addition, we used survminer package to explore the relationship between GGT5 expression and prognosis in TCGA database. We split patients into two groups by median value. All *P* values less than 0.05 were considered statistically significant.

### 2.6. Protein-Protein Interaction (PPI) Network Construction

In this research, STRING was applied (http://string-db.org) to analyze the coordinated protein control of GGT5 and the mutual coordinate function among points [18]. The mutual particularity rate  >0.4 was regarded as playing an important role.

### 2.7. Gene Set Enrichment Analysis (GSEA)

We used GSEA to show obvious diversity between the different performances of the GGT5 expression (version 4.1.0). Each analysis was repeated 1000 times to arrange the gene set, and the expression level of GGT5 was used as a phenotypic marker. If the *P* value showed no more than 0.05 and the data was lower than 0.25 of FDR, the gene group was thought to play an important role.

### 2.8. Tumor Immune Infiltration Analysis

All patients were divided into high- and low-expression groups (high-risk group and low-risk group) according to the median expression of GGT5. We then adopted Cell-type Identification by Estimating Relative Subsets of RNA Transcripts (CIBERSORT) method to qualify and quantify 22 types of immune cells between high-risk and low-risk groups [19]. We used the R software ESTIMATE algorithm to calculate the ESTIMATEScore, ImmuneScore, and StromalScore for each tumor sample and determined whether there were differences between the two groups. We also calculated differences in the expressions of PD1 (PDCD1), PDL1 (CD274), and CTLA4 in the high-risk and low-risk groups.

### 2.9. Statistical Analysis

R (version 4.0.3) was applied for data research. Wilcoxon test was performed to make a difference between GGT5 performance in stomach cancer and common case. We studied the connection between clinical samples and GGT5 in depth and used univariate and multivariate analysis to explore its possibility as an element and indicator of judgement. *P* < 0.05 is regarded as playing an important role in data.

## 3. Results

### 3.1. Searching DEGs between Tumor Tissues and Normal Tissues in Gastric Cancer

**Figure 1:**
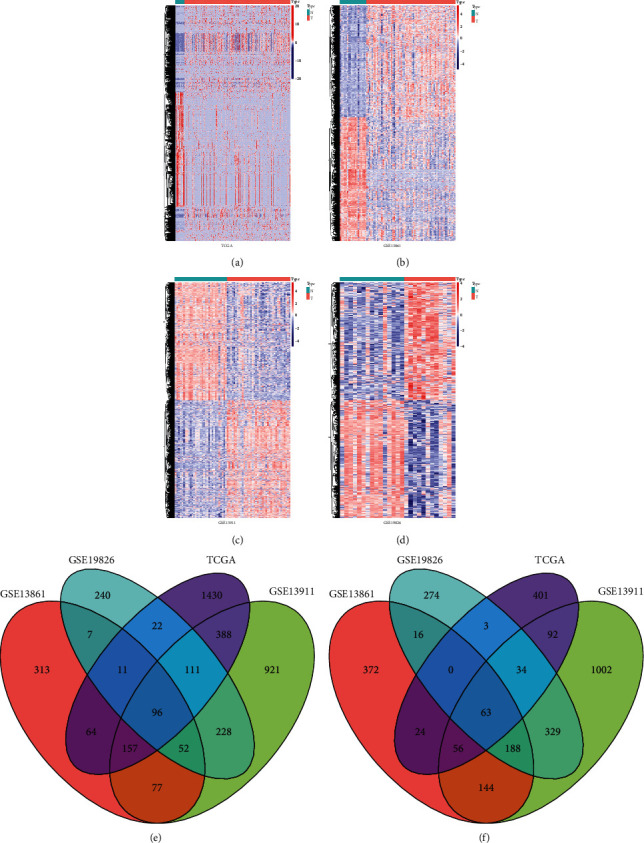
Differentially expressed genes between tumor tissue and normal tissue in gastric cancer. (a) Differential genes in the TCGA database. (b) Differential genes in GSE13861. (c) Differential genes in GSE13911. (d) Differential genes in GSE19826. (e) Venn diagram of upregulated genes. (f) Venn diagram of downregulated genes.

There are differences in morphology and physiological function between tumor tissue and normal tissue. In order to understand the causes of these differences, differential gene analysis was performed on four data sets and the intersection was taken. These four data sets are TCGA, GSE13861, GSE13911, and GSE19826. Due to the large number of differential genes obtained in TCGA, the threshold value was set as the adjust. *P* < 0.05 and |FC|  > 2, and the threshold value was set as the adjust. *P* < 0.05 and |FC|  > 1.5 in the other three GEO data sets. As shown in Figure 1(a), 2,952 differential genes were selected from the TCGA database, of which 2,279 genes were upregulated and 673 genes were downregulated in tumor tissues. In GSE13861, 777 genes were upregulated in tumor tissues and 863 genes were downregulated in tumor tissues (Figure 1(b)). There are 2,030 upregulated genes in tumor tissue and 1,908 downregulated genes in GSE13911 (Figure 1(c)).  Figure 1(d) shows that, in GSE19826, 767 genes were upregulated and 907 genes were downregulated in tumor tissues. Then, by taking the intersection of the differential genes in the four data sets, we found that 96 genes (Figure 1(e)) were upregulated and 63 genes (Figure 1(f)) were downregulated. Then we further searched for these differential genes on PubMed, and we found that GGT5 was upregulated in gastric cancer in these data sets, and few people had studied it. We thought that GGT5 might be a new marker to judge tumor prognosis.

### 3.2. Transcriptional Levels of GGT5 in Different Types of Cancers

**Figure 2:**
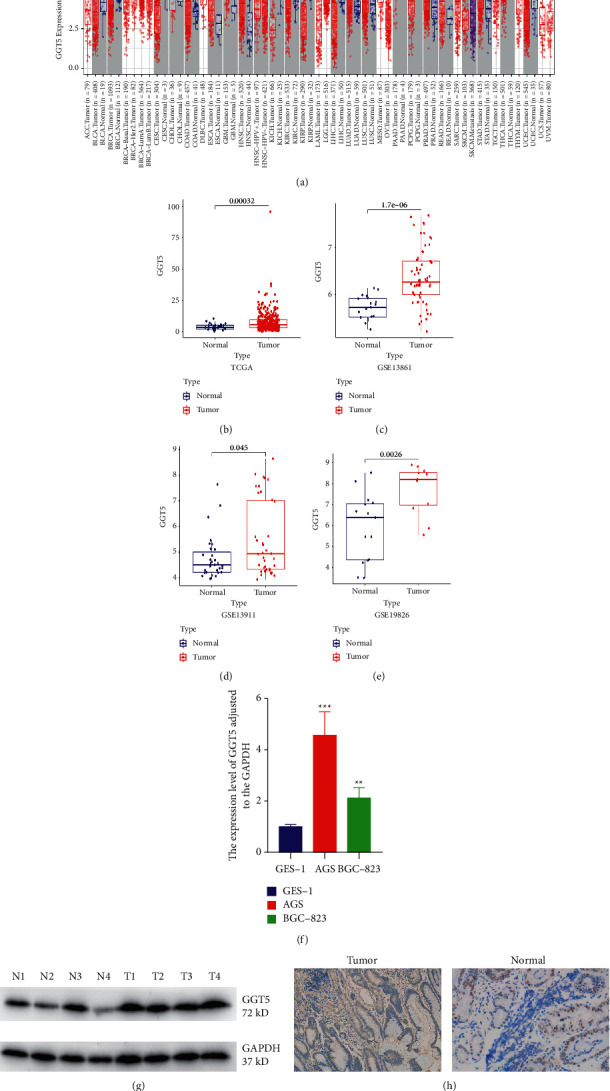
Transcriptional levels of GGT5 in cancers. (a) Transcriptional levels of GGT5 in different types of cancers. (b) GGT5 expression was significantly higher in cancer tissues than in normal tissues in TCGA. ((c)–(e)) GGT5 expression was significantly higher in gastric cancer tissues than in normal tissues in GSE13861, GSE13911, and GSE19826. (f) qPCR of gastric cancer cell lines and normal cell line. (g) WB of gastric cancer tissue and normal tissue. (h) IHC of gastric cancer tissue and normal tissue.

To evaluate the difference of GGT5 between diverse tumor and common cases, according to to TIMER2.0, we made a difference in expression results in the performance of TCGA. In Figure 2(a), GGT5 was highly expressed in ESCA (esophageal carcinoma), GBM (glioblastoma multiforme), LUAD (lung adenocarcinoma), PRAD (prostate adenocarcinoma), and STAD (stomach adenocarcinoma) compared with adjacent normal tissues. Furthermore, GGT5 was highly expressed in HPV-HNSC+ and SKCM (skin cutaneous melanoma) metastasis. However, GGT5 expression was significantly lower in PCPG (pheochromocytoma and paraganglioma), BRCA (breast invasive carcinoma), KICH (kidney chromophobe), KIRP (kidney renal papillary cell carcinoma), UCEC (uterine corpus endometrial carcinoma), KIRC (kidney renal clear cell carcinoma), and LIHC (liver hepatocellular carcinoma) making a difference from contiguous common parts.

Then, Wilcoxon rank-sum test was applied to give the research of GGT5 in different tissue characteristics, and the findings told us that GGT5 expression in gastric cancer tissues was significantly higher than that in normal tissues (Figure 2(b): *P* = 3.2 × 10^−4^). Subsequently, we used Wilcoxon-rank-sum test to detect the expression of GGT5 in tumor tissues and normal tissues in three GEO data sets. The results showed that the expression of GGT5 in tumor tissues was significantly higher than that in normal tissues (Figure 2(c): *P* = 1.7 × 10^−6^; Figure 2(d): *P*=0.045; Figure 2(e): *P*=0.0026). We also found that GGT5 expression was higher in gastric cancer cell lines than in normal cells (Figure 2(f): *P* < 0.05). We collected tissue specimens of gastric cancer patients from the hospital to further verify our results. WB results showed that the expression of GGT5 in gastric cancer tissues was significantly higher than that in normal tissues (Figure 2(g)). The densitometry readings/intensity ratio of each band is shown in Supplementary Figure 1. In addition, we observed the same result in immunohistochemistry (Figure 2(h) and Supplementary Figure 2).

### 3.3. Prognostic Value of GGT5 in STAD

**Figure 3:**
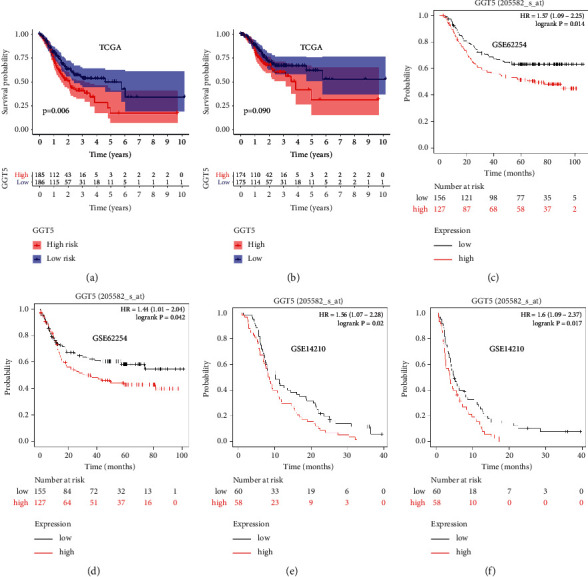
The prognostic value of GGT5 in STAD. (a) High expression of GGT5 was associated with poor OS in the TCGA database. (b) High expression of GGT5 was not associated with poor RFS in the TCGA database. (c) The probe associated with GGT5 was associated with poor OS in GSE62254. (d) High expression of GGT5 was associated with poor PFS in GSE62254. (e) The probe associated with GGT5 was associated with poor OS in GSE14210. (f) High expression of GGT5 was associated with poor PFS in GSE14210.

The findings presented that, compared with patients with low expression, high-expression GGT5 patients had shorter overall survival (OS) (Figure 3(a): *P*=0.02). We evaluated the prognostic value of GGT5 on relapse-free survival (RFS) in TCGA. We split patients by median value into two groups. We found that high GGT5 expression was not associated with poor RFS (Figure 3(b): *P*=0.09). Furthermore, we used KM Plotter database to evaluate the prognostic value of GGT5 based on GSE62254. As shown in Figure 3(c), high expression of GGT5 (probe 205582_s_at) was associated with poor OS (HR = 1.57, 95% CI = 1.09–2.25, log-rank *P*=0.014). The same result was observed in progression-free survival (PFS) (Figure 3(d), HR = 1.44, 95% CI = 1.01–2.04, log-rank *P*=0.042). We further explored the prognostic value of GGT5 in the GSE14210 data set. The results showed that high expression of GGT5 (probe 205582_s_at) was associated with poor OS (HR = 1.56, 95% CI = 1.07–2.28, log-rank *P*=0.02) and PFS (HR = 1.6, 95% CI = 1.09–2.37, log-rank *P*=0.017).

### 3.4. Interaction Networks of GGT5

**Figure 4:**
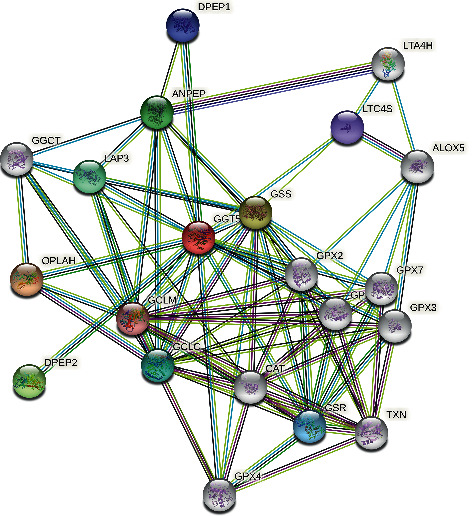
The network analysis of GGT5.

To further study the molecular mechanism of GGT5 genes in tumorigenesis, the targeted binding protein of GGT5 was tried to be screened out to perform a variety of pathway enrichment analysis. Based on the STRING website, 20 GGT5 binding proteins were acquired. The network of interactions between these proteins is presented in Figure 4.

### 3.5. Relationship between GGT5 and the Clinicopathological Parameters of Patients with STAD

**Figure 5:**
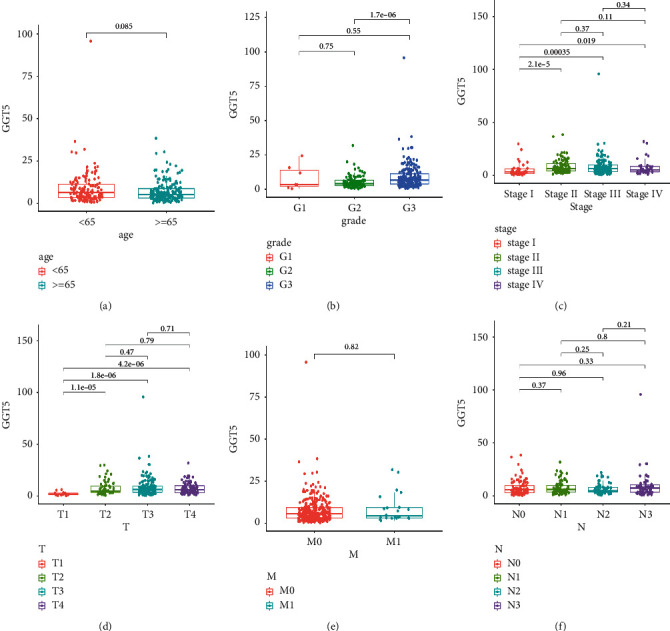
Relationship between GGT5 expression and clinicopathological characteristics. (a) Age. (b) Grade. (c) Stage. ((d)–(f)) TNM classification. T, topography distribution; N, lymph node metastasis; M, distant metastasis.

To better understand the correlation and potential mechanism of the expression of GGT5 in cancer, we studied the relationship between the expression of GGT5 and the clinical characteristics of gastric cancer patients in the TCGA database. According to the expression levels of GGT5, we divided the patients into two groups with high and low expression to explore the correlation between GGT5 and clinical characteristics. Chi-square test and *T* test were used for data analysis, and the results are shown in Table 1. We found that GGT5 expression was associated with age (*P*=0.049), T stage (*P*=0.001), and pathologic stage (*P*=0.002). The result of Wilcoxon rank-sum test revealed that the upregulation of GGT5 was related to grade, stage, and T stage, as shown in Figure 5. The result showed that GGT5 was highly expressed in grade 3 (Figure 5(b)) and was highly expressed in stages II, III, and IV (Figure 5(c)). We also found that the expression of GGT5 was higher in stages T2, T3, and T4 (Figure 5(d)). The expression level of GGT5 was not correlated with other clinical traits such as age, N stage, and metastasis.

### 3.6. GGT5 May Be an Independent Predictor of Prognosis in STAD

**Figure 6:**
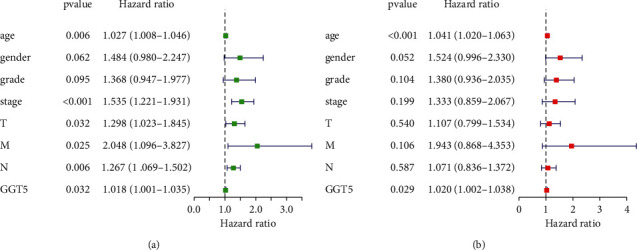
Univariate analysis and multivariate analysis of GGT5 in STAD. (a) A forest map of the results of the univariate analysis. (b) A forest map of the results of the multivariate analysis. T, topography distribution; N, lymph node metastasis; M, distant metastasis.

To further verify the effect of GGT5 on prognosis and to evaluate the prognostic variables related to OS (Figure 6), univariate and multivariate Cox regression analyses were used by us. According to the univariate Cox model, there was a close correlation between the high expression of GGT5 and the deterioration of OS (Figure 6(a): HR = 1.018; 95% CI: 1001–1.035; *P*=0.032). High expression of GGT5 was the independent factor of prognosis related to OS (HR: 1.020; 95% CI: 1.002–1.038; *P*=0.029) as presented in Figure 6(b), in the multivariate analysis.

### 3.7. GSEA Analysis

**Figure 7:**
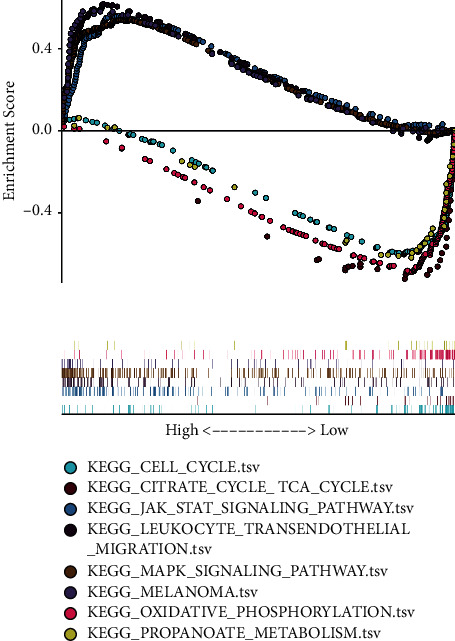
Enrichment plots from the gene set enrichment analysis (GSEA).

We used GSEA to analyze the pathways that there was a remarkable distinction in the data set between GGT5 high expression and low expression. The results showed that overexpression of GGT5 might be involved in cancer-related pathways, such as JAK-STAT and MAPK signaling pathway, leukocyte transendothelial migration, and melanoma. Low expression of GGT5 might be involved in cell cycle, citrate cycle TCA cycle, oxidative phosphorylation, and propanoate metabolism pathway (Figure 7).

### 3.8. The Infiltrating Immune Cells in STAD

**Figure 8:**
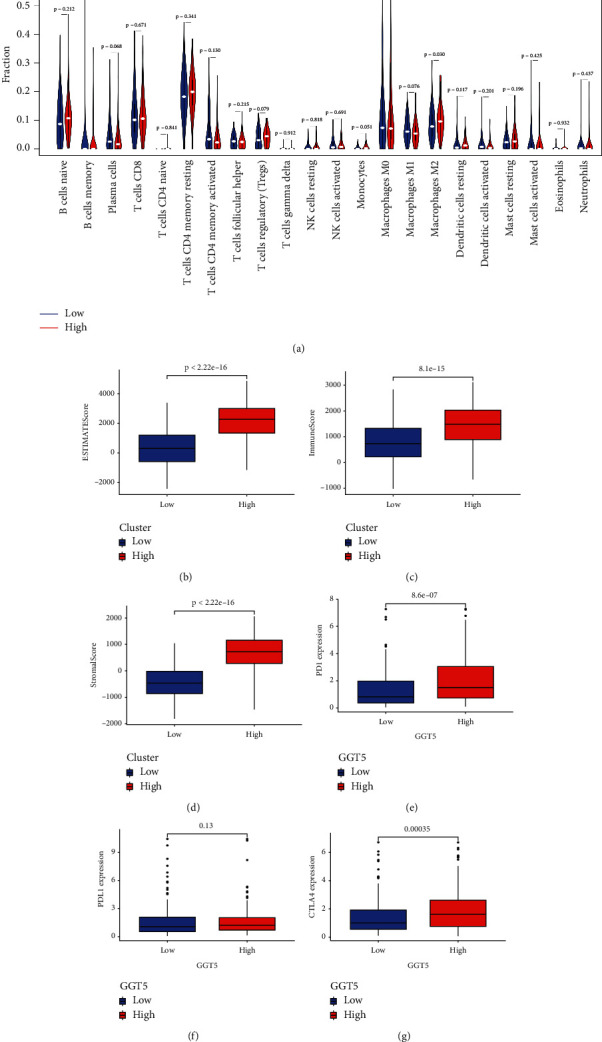
The infiltrating immune cells in STAD. (a) The morphology of infiltrating immune cells between two groups in STAD. ((b)–(d)) The ESTIMATEScore, ImmuneScore, and StromalScore between the high-expression and low-expression GGT5 groups. ((e)–(g)) The expression levels of three immune checkpoints between the two groups.

Recently, immunotherapy, as a new therapeutic method, has achieved good results in the treatment of many tumors [20]. We extracted and processed characteristic gene expression profiles of immune cells using CIBERSORT method and systematically described the patterns of immune cells. After removing the samples with *P* ≥ 0.05, the morphology of infiltrating immune cells between two groups in STAD is shown in Figure 8(a). The results showed that the concentration of M2 macrophages was higher in the group with high GGT5 expression (*P*=0.03). Studies have shown that M2 macrophages are related to tumor progression [21], which explains why the high-expression GGT5 group has a worse prognosis. At the same time, we found higher levels of T cell regulators (Tregs) (*P*=0.079) and monocytes (*P*=0.051) in the high-expression GGT5 group, but *P* values were not significant. We also found that the contents of plasma cells (*P*=0.068) and M1 macrophages (*P*=0.076) were higher in the low-expression GGT5 group, and the *P* value was not significant. To further verify the immune infiltration between the high-expression and low-expression GGT5 groups, ESTIMATEScore, ImmuneScore, and StromalScore were calculated for each tumor sample, respectively. The results showed that the ESTIMATEScore, ImmuneScore, and StromalScore of high-expression GGT5 group were higher than those of low-expression GGT5 group (Figures 8(b)–8(d); all *P* ≤ 0.05). This suggested that the high-expression GGT5 group had a higher degree of immune cell infiltration, which might be more sensitive to immune checkpoint therapy. We then explored the expression levels of three immune checkpoints between the two groups. The results showed that the expression levels of PD1 and CTLA4 (Figures 8(e) and 8(g); all *P* ≤ 0.05) in the high-expression GGT5 group were significantly higher than those in the low-expression GGT5 group, which further confirmed our results that the high-expression GGT5 group may be more effective for immunotherapy.

## 4. Discussion

Although significant progress has been made in diagnosis, intervention, and therapy, the prognosis of these patients remains poor, and the survival rate is not ideal [22]. Molecular biomarkers, as prognostic and diagnostic features are increasingly developed and applied in clinical practice, have greatly facilitated patient classification, disease state monitoring, and personalized treatment schedules [23]. In recent years, more and more evidence has shown that the increase of serum GGT is involved in the occurrence and development of tumors, like gastric cancer [24], primary hepatic carcinoma [25], colorectal cancer [26], and cervical cancer [27]. However, the role of GGT5 in tumorigenesis remains unclear. At present, the prognostic value of GGT5 in STAD is our focus.

In this study, it is found that, compared with normal tissues, GGT5 had a high expression in many tumor tissues. Observation of gastric cancer also yielded the same result. Moreover, clinical samples had further confirmed our results. In addition, high GGT5 in STAD tissue predicted patients' progression-free survival rate and poor overall survival rate, and these results are consistent with the conclusions of two other papers [28,29]. These findings suggest that GGT5 exerts a positive function in STAD progression, but its function and mechanism remain unclear. Another study in lung cancer has shown that the gene GGT5 is highly expressed in cancer-associated fibroblasts (CAFs) in lung adenocarcinoma, and high levels of GGT5 contribute to the survival and drug resistance of cancer cells in CAFs [12]. However, we need further experimental studies to verify our conclusions.

According to the AJCC Staging Manual for Gastric Cancer, the later a patient's tumor stage is, the worse the prognosis is [30]. It was discovered that there is a high expression of GGT5 in grade 3, stages T2, T3, and T4, and stages II, III, and IV. This further explains why the high expression of GGT5 is correlated to a bad prognosis. Then, we conducted a univariate and multivariate analysis of the influence of GGT5 on the prognosis. The results showed that GGT5 could predict patient outcomes independently of other clinical characteristics.

In this study, we used GSEA to analyze possible significant enrichment pathways in the high-expression group of GGT5. The results showed that overexpression of GGT5 might be involved in signaling pathway of JAK-STAT leukocyte, as well as MAPK, transendothelial migration, and melanoma pathway. Recent studies have shown that there is a clear correlation between these pathways and the progression of cancer [31–34]. As a result, GGT5 could promote the growth of gastric cancer cells by considering these results and tumor metastasis through the above signaling pathways, leading to poor survival of gastric cancer patients, and its specific regulatory mechanism needs further experimental study.

With the development of tumor research, people gradually realize that tumor microenvironment plays an important role in tumor. Cancer cells promote tumor growth and development by promoting blood and lymphatic formation and immune suppression through their constant interaction with the surrounding environment [35]. Our study showed that the GGT5 overexpression group had higher concentrations of M2 macrophages, T cell regulators, and monocytes, which have been shown to be associated with poor prognosis [36–38]. This is consistent with our conclusion. We also found that the contents of plasma cells and M1 macrophages were higher in the low-expression GGT5 group. Studies have shown that M1 macrophages are associated with good prognosis [39]. This further supports our conclusion. We also found that the ESTIMATEScore, ImmuneScore, and StromalScore of the high-expression GGT5 group were higher than those of the low-expression GGT5 group. We then explored the expression levels of three immune checkpoints between the two groups. The results showed that PD1 and CTLA4 expression levels were higher in the high-expression GGT5 group. Further confirming our results, the group with high GGT5 expression may be more effective for immunotherapy.

The potential prognostic function of GGT5 in STAD was explored for the first time and presented in our study. Nevertheless, our study still has some limitations. One limitation is that our study lacked a control group, and there could be a potential bias. The data was collected in different laboratories, resulting in inconsistent data collection processes or insufficient information in this study, which is another limitation of this study. Therefore, it is necessary to conduct further experiments to investigate the detailed mechanism of GGT5 in gastric cancer.

## 5. Conclusions

In short, we concluded that there is an elevated expression level of GGT5 in gastric cancer tissues, which was related to advanced tumor stage and poor prevision. Overexpression of GGT5 might be involved in cancer-related pathways, such as signaling pathway of JAK-STAT, as well as transendothelial migration of leukocyte, melanoma, and MAPK signaling pathway. The high-expression GGT5 group had higher concentrations of M2 macrophages, T cell regulators, and monocytes, but the contents of plasma cells and M1 macrophages were higher in the low-expression GGT5 group. PD1 and CTLA4 expression levels were higher in the high-expression GGT5 group. The group with high GGT5 expression may be more effective for immunotherapy. These results suggested that they might be factors in STAD patients used to predict potential prognostic molecules. The outcome added new ideas to the treatment of gastric cancer.

## Figures and Tables

**Table 1:** Relationship between expression of GGT5 and clinicopathological characteristics.

Characteristic	Low expression of GGT5	High expression of GGT5	*P*
*n*	187	188	
*T stage, n (%)*				
T1	17 (4.6%)	2 (0.5%)	<0.001
T2	48 (13.1%)	32 (8.7%)	
T3	73 (19.9%)	95 (25.9%)	
T4	45 (12.3%)	55 (15%)	

*N stage, n (%)*				
N0	59 (16.5%)	52 (14.6%)	0.173
N1	48 (13.4%)	49 (13.7%)	
N2	42 (11.8%)	33 (9.2%)	
N3	29 (8.1%)	45 (12.6%)	

*M stage, n (%)*				
M0	166 (46.8%)	164 (46.2%)	1.000
M1	13 (3.7%)	12 (3.4%)	

*Pathologic stage, n (%)*				
Stage I	39 (11.1%)	14 (4%)	0.002
Stage II	50 (14.2%)	61 (17.3%)	
Stage III	67 (19%)	83 (23.6%)	
Stage IV	21 (6%)	17 (4.8%)	

*Gender, n (%)*				
Female	69 (18.4%)	65 (17.3%)	0.718
Male	118 (31.5%)	123 (32.8%)	

*Age, n (%)*				
≤65	71 (19.1%)	93 (25.1%)	0.049
>65	112 (30.2%)	95 (25.6%)	
Age, median (IQR)	69 (59, 75)	66 (57, 72)	0.013

## Data Availability

The data of this study are available from the corresponding author upon request. The gene expression profile is based on TCGA and GEO data sets.

## Supplementary Materials

Supplementary Materials The densitometry readings/intensity ratio of each band is shown in Supplementary Figure 1. The result of immunohistochemistry is shown in Supplementary Figure 2.
