# Supplementary figures and images for: GGT5 Is an Independent Prognostic Biomarker in Stomach Adenocarcinoma
Source: Can J Gastroenterol Hepatol. 2022 Feb 26;2022:9983351. doi: 10.1155/2022/9983351 (PMC8898138; doi:10.1155/2022/9983351)

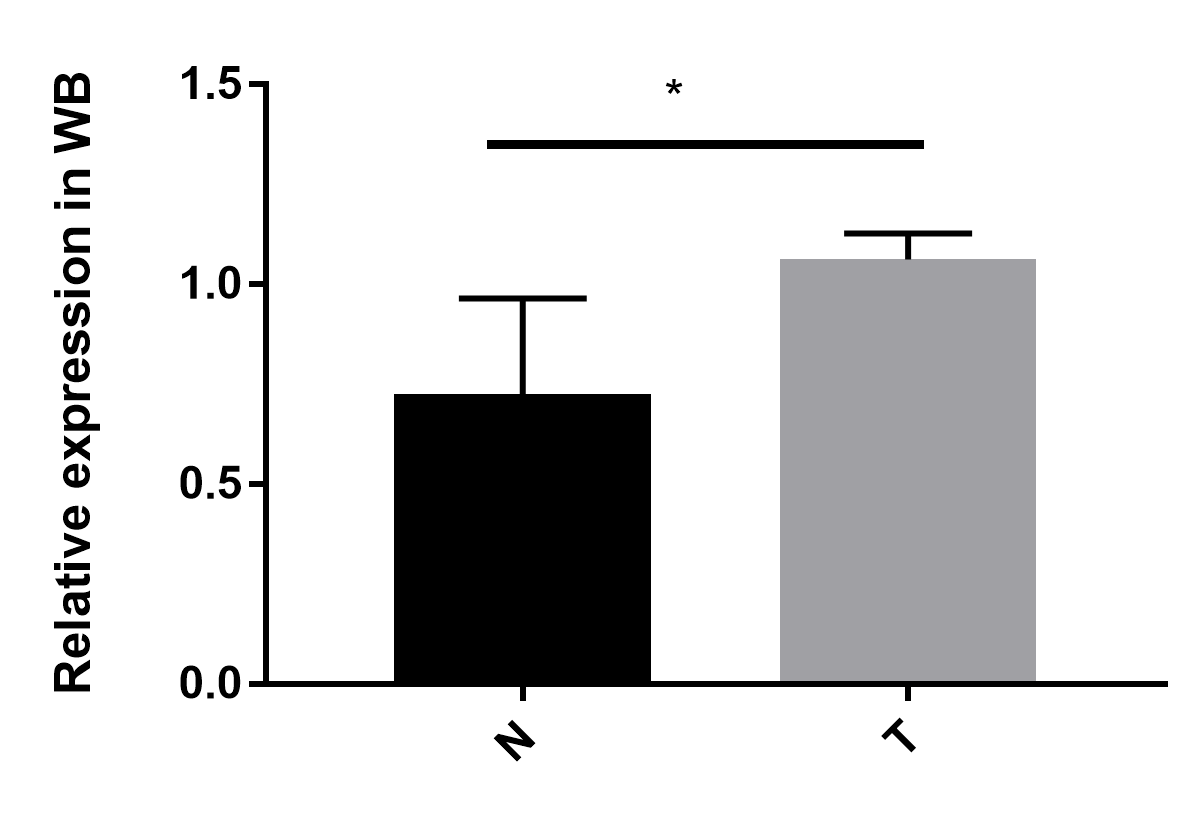


sup Figure 1


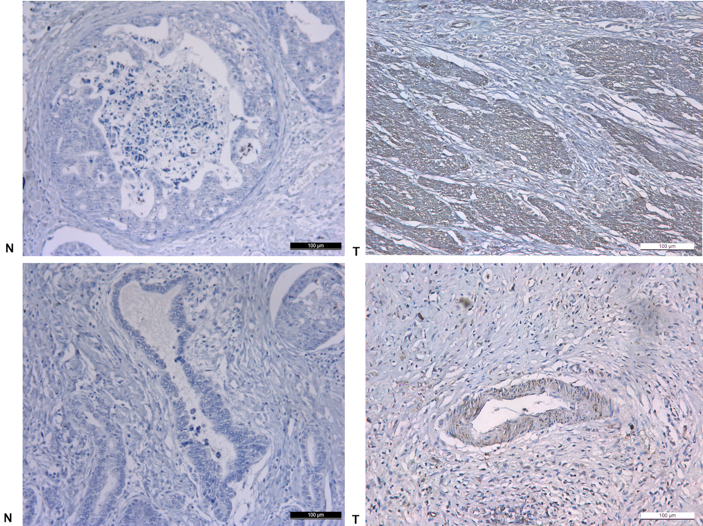


sup Figure 2

Supplement: Supplementary Materials — The densitometry readings/intensity ratio of each band is shown in Supplementary Figure 1. The result of immunohistochemistry is shown in Supplementary Figure 2. [file 9983351.f1.docx]
